# Niche Construction on Environmental Gradients: The Formation of Fitness Valley and Stratified Genotypic Distributions

**DOI:** 10.1371/journal.pone.0099775

**Published:** 2014-06-10

**Authors:** Xiaozhuo Han, Cang Hui

**Affiliations:** 1 School of Applied Mathematics, Guangdong University of Technology, Tianhe District, Guangzhou, China; 2 Centre for Invasion Biology, Department of Mathematical Sciences, University of Stellenbosch, Matieland, South Africa; 3 Mathematical and Physical Biosciences, African Institute for Mathematical Sciences, Muizenberg, South Africa; Virginia Tech, United States of America

## Abstract

The process of niche construction can alter the trajectory of natural selection through organism-environment feedback. As such, the mechanism and impact of niche construction can be better investigated along environmental gradients. Here we investigate how the process of niche construction affects the distribution of genotypes and fitness landscape along an environmental gradient under three selection regimes, namely heterozygote superiority, genetic loci which dictates niche construction ability being either selectively neutral or non-neutral. Using a spatially explicit cellular automaton, we show that niche construction can stratify genetic diversity by forming band-like distributions consisting of different genotypic compositions and promote reproduction isolation by forming a divide with reduced average fitness along the gradients, termed a fitness valley. The band structure and the presence of a fitness valley depend on heterogeneous environments, resource-dependent fitness and the selection acting on the gene loci affecting the niche-constructing ability. Our work adds to the growing body of evidence on criticizing species distribution models which assume that the environment alone can determine species distributions. Based on the results, we argue that conservation planning should target preserving or restoring environmental gradients.

## Introduction

Organisms are not passively selected by their ambient environment but coevolve with their environment through either direct niche construction (also called ecosystem engineering; e.g. digging burrows and spreading webs) or indirect life-history activities (e.g. photosynthesis in plants that can fix atmospheric carbon into the soil and thus dramatically alter the soil profile) [1]–[6]. The process of niche construction can form a positive or amplified feedback between the demand and supply of limiting resources and can, thus, potentially affect the evolutionary trajectory by modifying the selective pressure of certain traits, especially those that are responsible for the niche construction [7]–[8]. Indeed, the concept of niche construction emphasizes the importance of organism-environment coupling during adaptive evolution and has been suggested to be capable of promoting stable polymorphism [9], altering competition outcomes [10]–[11], fixing deleterious alleles [12]–[15], and posing evolutionary momentum of directional selection [16]–[17]. However, studies on the effect of niche construction on the spatial distribution of species and their genetic structures are lacking, especially regarding how the process of niche construction can affect the distribution of different genotypes and how it can potentially promote the formation of range boundaries which further promote diversification and polymorphism.

The fitness of an organism is a rather context-based term, depending on whether its niche requirement matches the characteristics of inhabited environment. A highly fit genotype in one environment does not warrant a high fitness in other environments. Consequently, species often exhibit large variation of morphological traits and life-history strategies at regional scales (e.g. [18]–[20]). Such variation is maintained by both genetic and environmental factors and often shows a systematic shift along environmental gradients (e.g. along rainfall or altitudinal gradients; [21]–[24]). To this end, studies along environmental gradients become particularly appealing because they provide an ideal experiment for examining how the coupling of genetic structures and environmental factors interact to affect fitness along the environmental gradient. This further helps to resolve the longstanding debate on the role of genetic variation in species' adaptation to environmental heterogeneity [25]. It is thus important to further assess how the process of niche construction affects the genetic structure and fitness landscape along environmental gradients.

We here investigate how the process of niche construction interacts with resources along an environmental gradient and how it further affects genetic structures and polymorphism patterns. Using a spatially explicit model of population genetics on lattices, we demonstrate the pattern of genetic diversity and population fitness along the environmental gradient. Specifically, two questions on how population genetics and fitness landscape change along environmental gradients are addressed. First, species distribution models (also known as ecological niche modelling) have been widely used in conservation management to project the potential distribution of a focal species or genotype based on detected relationships between species observed distribution and environmental characteristics (e.g. [26]–[29]). An underlying assumption of such models is that environment dictates species distribution. That is, population density and genotypic frequency change gradually in response to the change of resources along environmental gradients, without clear boundaries. In other words, clear boundaries of species distributions should reflect sudden change in environmental resources or dispersal barriers. Although this top-down effect of environmental filtering on species distribution can be strong at regional scales [30]–[32], it does not explain the common observation of clear distributional boundaries of species and genotypes at local scales even if the environmental gradient is subtle or continuously changing. We here demonstrate how the process of niche construction affects the formation of clear distributional boundaries along a linear environmental gradient.

Second, to promote diversification and polymorphism in adaptive evolution, species need to possess certain reproduction barriers after disruptive selection to prohibit remixing [33]–[35]. This reproduction barrier can be set up either via assortative behaviors (e.g. [23], [36]) or a physical barrier (e.g. mountains and rivers as dispersal barriers; [37]). Can the process of niche construction along a linear environmental gradient promote the formation of a reproduction barrier? We here examine the potential of a fitness valley (reproductive barrier) driven by niche construction in the fitness landscape along a linear environmental gradient that separates species distributions and stratifies species genetic structures along the environmental gradient. This fitness valley could restrict potential gene flows and function as a reproduction barrier during allopatric diversification.

### Model

To examine the spatial distributions of different genotypes that differentially affect local nutrient content via the process of niche construction along a linear environmental gradient, we built a spatially-explicit individual-based cellular automaton (CA) on 200×200 lattices, where each cell contains a random-mating diploid individual with two dialelic loci **E** and **A** [15]. We assume that the frequency of allele *E* at generation *t* (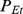) affects the individual's capacity of niche construction [13] and that the niche construction can affect the within-cell environmental resource positively or negatively by either producing or consuming the resource. Specifically, in each generation, the amount of resource (*R*) in a specific cell is governed by three processes (independent depletion, renewal and niche construction):



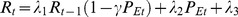
(1)


where 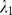 and 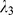 are coefficients of independent resource depletion and renewal; 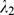 and 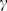 are coefficients of positive and negative niche construction. We assume 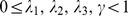 and 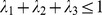, where the latter keeps the amount of resource within [0,1] interval. If there is no niche construction (i.e. 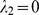 and 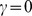), the resource will converge to a stable level (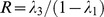). In the following, we ignore negative niche construction (i.e. 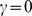).

Following Laland et al. [13], we assume that both genotypes at loci **E** and **A** contribute to a two-locus fitness, 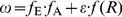
(2)


where 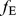 represents the fitness contribution from locus **E** ( =  *α*_1_ for genotype *EE*, 1 for *Ee* and *β*_1_ for *ee*); 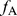 represents the fitness contribution from locus **A** ( =  *α*_2_ for genotype *AA*, 1 for *Aa* and *β*_2_ for *aa*); the coefficient 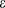 (−1<
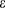
<1) determines the strength of the resource-dependent component (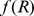) relative to the fixed-fitness component (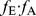).We consider three selection regimes: heterozygote advantage (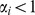 and 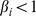), selection only at the ***A*** locus (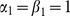), and selection only at the ***E*** locus (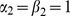). As above, the resource level was affected by the frequency of allele *E*; in return, the resource level then affects the individual fitness (Eq. (2)) by the additive term (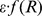) and interferes the fitness at locus **A** (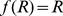 for *AA*, 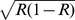 for *Aa* and 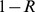 for *aa*) (Laland et al., 1999).

In the individual-based CA model, we introduced a linear environmental gradient along the vertical direction of the lattices (increasing from bottom to top). Specifically, we assume that the coefficient of independent resource renewal (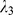) is not a constant but a linear function of the vertical coordinates (*y*) of the cell, 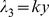, where *k* can be considered an indicator of the gradient of the environmental resource. To ensure the resource ranges from 0 to 1 along *y* axis we let 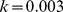 in the following analysis.

We chose periodic boundaries for the left and right edges to diminish the boundary effect and reflective boundaries for the top and bottom edges. Each cell of the lattices was initially randomly assigned one of the nine genotypes. During each time step, the individual in a focal cell chose to mate with the individual having the highest fitness in the four nearest neighboring cells, and then the individual was replaced one of its offspring randomly chosen according to the following fitness-dependent probability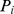: 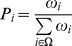
(3)


where 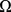 is the set of all possible genotypes that the parent can produce; 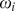 is the fitness of the 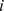 th genotype. The resource level (*R*) of this cell was then updated according to Eq.(1).

We used the Shannon *H* index to describe the genetic diversity for individuals on each row in the lattices [38]–[39],
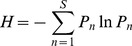
(4)


where *S* represents the number of genotypes and 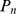 the proportion of genotype *n*. Shannon's *H* ranges from 0 to ln*S* and increases either when there is a high number of genotypes or when genotype frequencies are even. The minimum genetic diversity occurs when only one genotype exists (*H*  =  0), whilst the maximum genetic diversity occurs when the frequencies of all nine genotypes are equal (i.e. 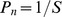 and *H*_max_  =  2.198).

## Results

**Figure 1:**
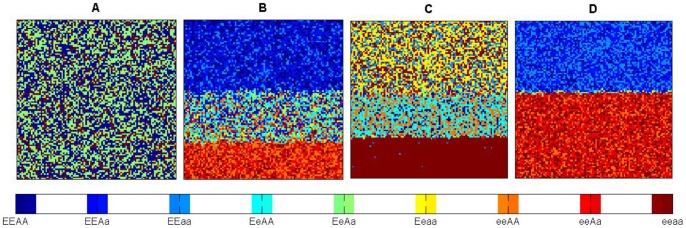
The distribution of genotypes on environmental gradients under three selection regimes. No environmental gradient in (A) where 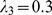; linear gradient in (B), (C) and (D), with 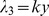, where 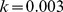 along *y* axis that having 100 coordinated points. Heterozygote superiority is assumed in (A) and (B), with 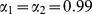, 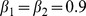; (C) selection only acts on locus *A*, with 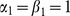, 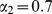, 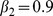); (D) selection only acts on locus *E*, with 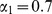, 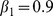, 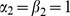. Other parameters are set in the cellular automaton: 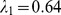, 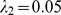, 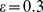
_._

**Figure 2:**
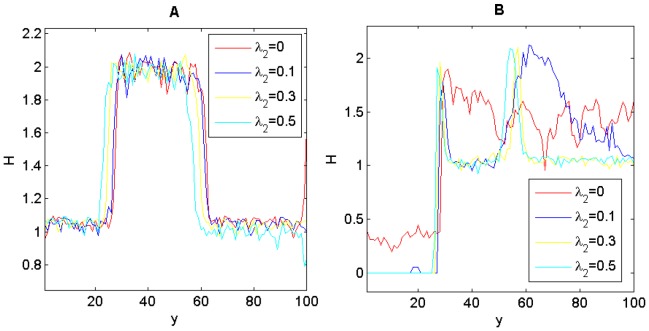
Genotypic diversity (*H*) as a function of the linear environmental gradient. Different lines show the change of genotypic diversity under different intensities of positive niche construction (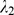). Plot (A) and (B) are for heterozygote superiority and selection on locus *A*, respectively. Other parameters are same as in Fig.1B & C.

When there was no environmental gradient, no clear patterns emerged (Fig.1A), contrasting to the clear patterns of triple-band (Fig.1B & C) and double-band distributions of genotypes (Fig.1D) along the environmental gradient. Genotypic diversity, as depicted by the Shannon *H* index, also showed a step-wise form (Fig.2), corresponding to the triple- and double-band distributions. With the increase of positive niche construction intensity (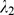), this step-wise form further shifted towards the direction of lower resources (Fig.2A), and the genotypic distribution was transferred from a double-band to a triple-band pattern (Fig.2B). Note that, even without niche construction (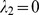), the environmental gradient can still stratify the genotypic distributions.

**Figure 3:**
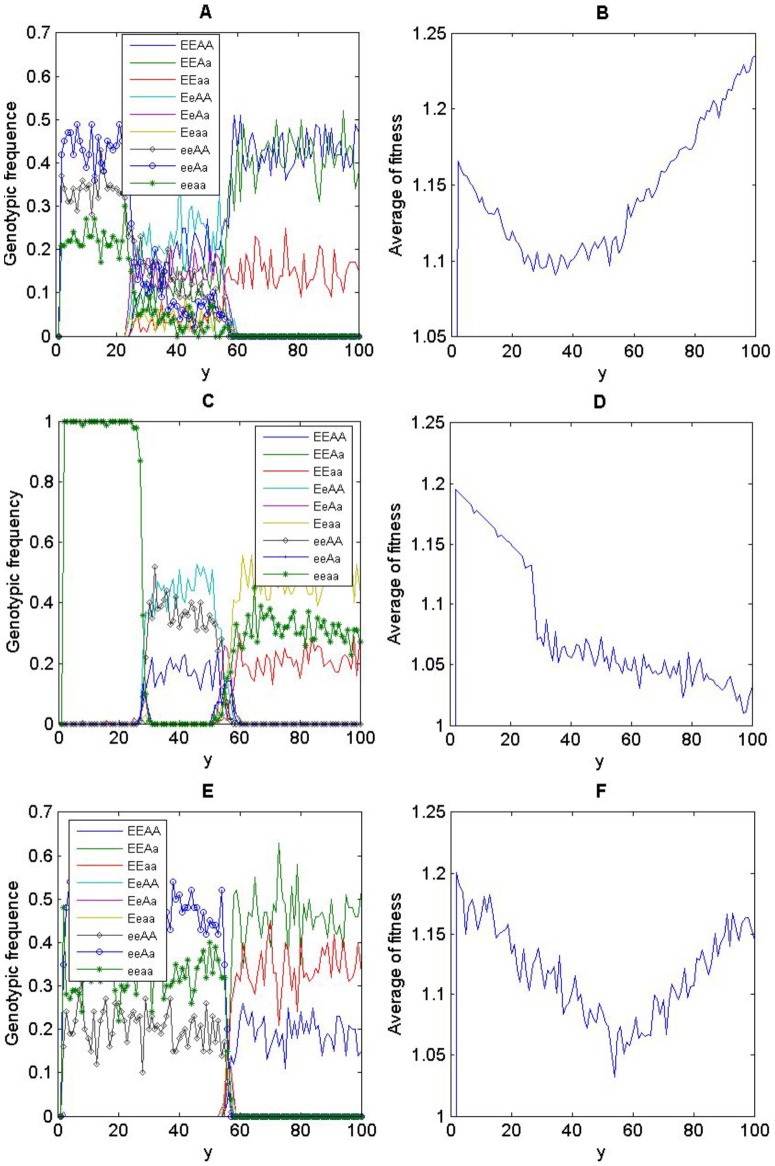
Genotypic frequency and average fitness as a function of the linear environmental gradient. Heterozygote superiority is assumed in (A) and (B); selection on locus *A* in (C) and (D); selection on locus *E* in (E) and (F). Parameters are the same as in Fig.1B, C & D.

The step-wise form of genotypic diversity suggests that the genotypic diversity reached its peak at an intermediate resource level along the environmental gradient. This is because the transition of genetic composition happens at the intermediate resource level, with one or three genotypes being completely replaced by another three genotypes towards the other end of the gradient (Fig.3A, C & E). When further examining the average fitness of the individuals on each row, we found that the fitness landscape along the environmental gradient formed a valley at the intermediate resource level where the transition of genetic composition occurred (Fig.3B & F). When the selection was not acting on the niche construction locus, the fitness valley was inconspicuous (Fig.3D), even though the transition of genetic composition still occurred (Fig.3C).

**Figure 4:**
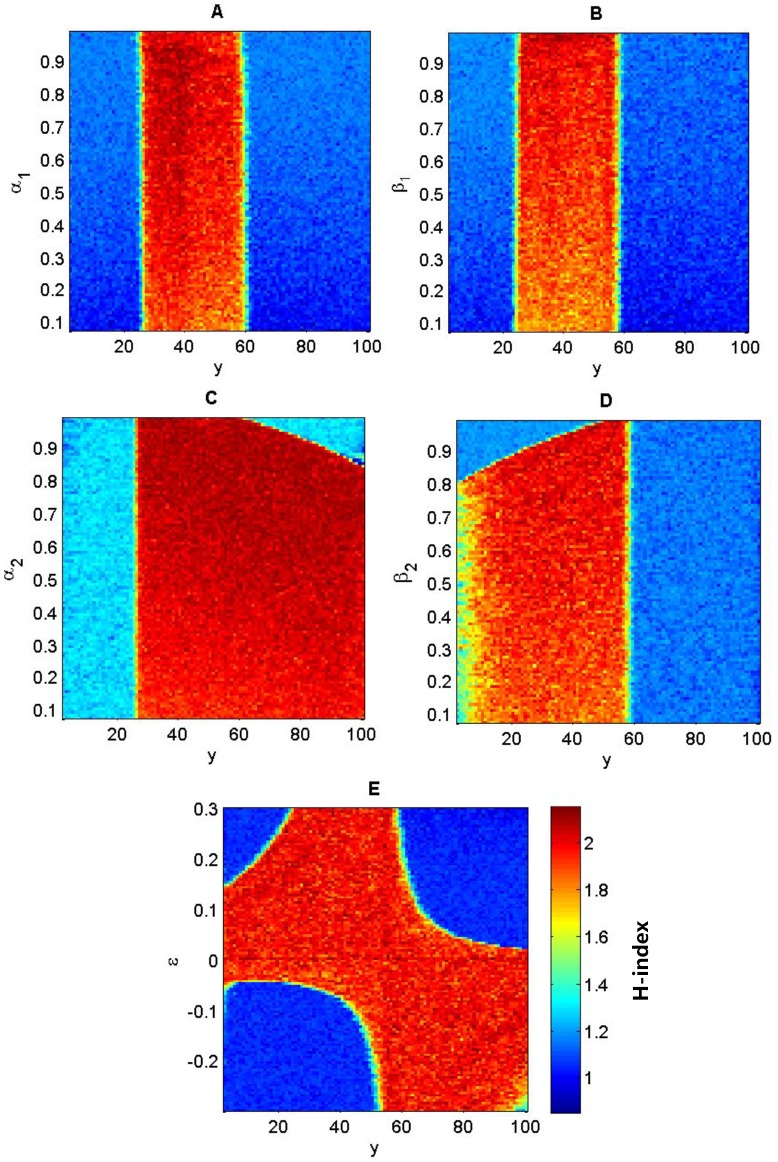
Genotypic diversity (*H*) as a function of the linear environmental gradient and fitness parameters (*α*, *β* and *ε*) under heterozygote superiority (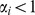 and 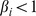). Other parameters are the same as in Fig.1B.

Both the intensity of selection (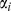 and 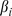) and the coefficient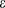 can affect the genotypic distribution. If individual fitness is independent from the resource level (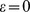), no band-like distribution of genotypes will emerge, regardless of the selection regimes. When the species experiences the selection regime of heterozygote superiority (i.e. 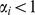 and 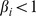, Fig.4), the effects of *α*_1_ and *β*_1_ on the genetic diversity *H* are rather similar (Figure 4A & B), showing the triple-band distribution that is independent from the selection intensity on locus ***E***. In contrast, with the increase of selection intensity on locus ***A***, the distribution of genotype changed from a double-band pattern (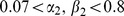) to a triple-band pattern (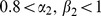) (Fig.4C & D). In addition, the double-band distribution of genotypes will emerge only if 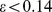, and a triple-band distribution will emerge only if 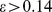 (Fig.4E). That is, the more affected the individual fitness is by the resource-dependent component relative to the fixed-fitness component, the more likely that the stratified distributions of genotypes will occur.

**Figure 5:**
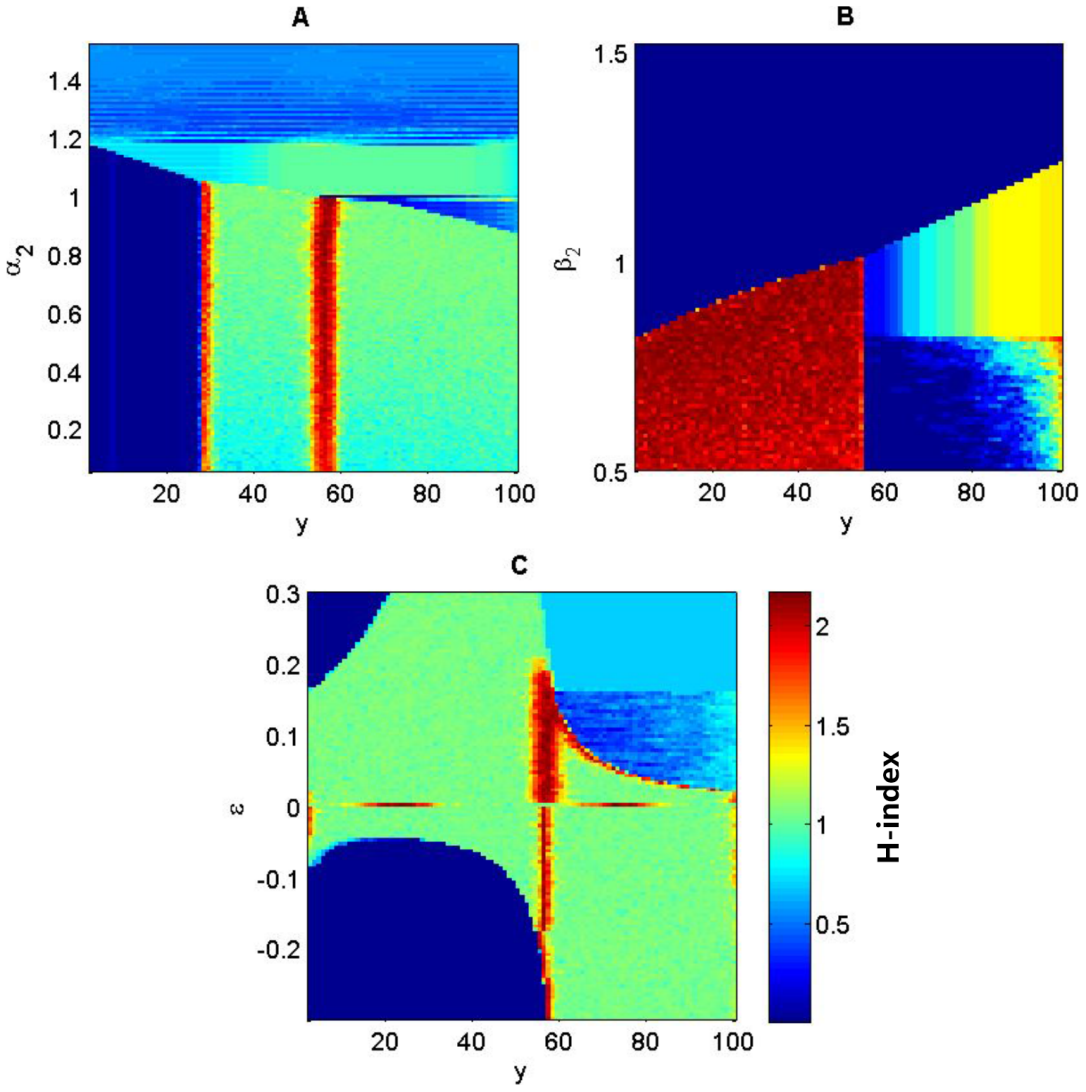
Genotypic diversity (*H*) as a function of the linear environmental gradient and fitness parameters (*α*, *β* and *ε*) when selection acts on locus *A* (i.e.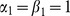). Parameters: 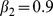 in (A); 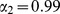 in (B), with other parameters 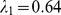, 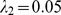, 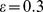; 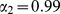 and 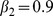 in (C).

When the selection acts on locus ***A*** (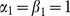,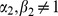, Fig.5), there is no stratified genetic diversity if 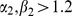; otherwise, the distribution of genotype would be hyper sensitive to the change of coefficient 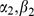
and hence forms double- or triple-band patterns (Fig.5A & B). The genetic diversity varying with the coefficient 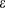
is similar to the selection regime of heterozygote superiority (Fig.5C, comparing with Fig.4E), showing a triple-band pattern when 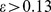 and a double-band pattern for other values of 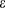.

**Figure 6:**
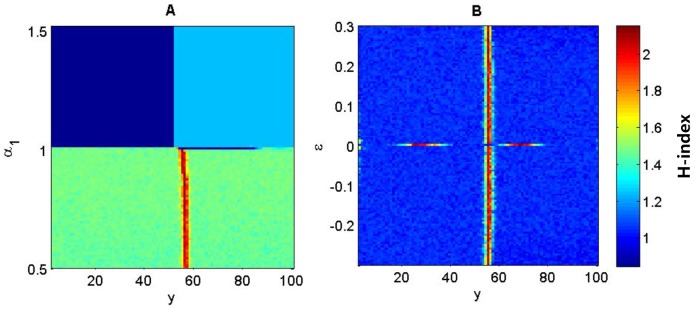
Genotypic diversity (*H*) as a function of the linear environmental gradient and fitness parameters (*α* and *ε*) when the selection acts on locus *E* (i.e. 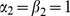). Parameters: 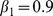 and 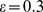
in (A); 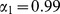 and 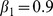 in (B); others 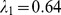, 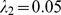.

When the selection acts on locus ***E*** (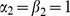, 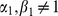, Fig.6), there are three genotypes in each band when 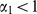 (Fig.6A), with the transition between bands occurring instantly (referring to Fig.3E). Only one genotype (eeAA) exists in the lower half of the environmental gradient if 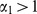, (Fig.6A). The sensitivity of genotype distributions to coefficient *β*_1_ is similar to the sensitivity to coefficient *α*_1_ and is thus not shown. As the alleles on locus ***E*** affect both the resource level through niche construction and the fitness, the genetic diversity *H* becomes insensitive to the relative contribution (*ε*) of the resource-dependent component to the overall fitness, except for the extreme case when 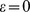 (Fig.6B). Each band consists of three genotypes when 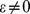 and switches to another three genotypes instantly at the middle point of the environmental gradient (Fig.6B).

## Discussion

The concept of niche construction emphasizes the change that organisms bring about in their selective environments and is considered an evolutionary process rather than an evolutionary product [40]. The effects of niche construction can, arguably, persist over geological time, modulating macro-evolutionary patterns and species diversity [4], forming the momentum of evolution towards specific directions [12], [16]. Niche construction can accelerate the formation of steady polymorphism especially under deteriorating habitats and thus impede the negative impact of harmful environments [15]. For instance, the adaptive feedback between plants and their soil environment could account for why plants partially regulate soil nutrient content and thus possess evolutionary advantage during ecological succession and species packing [41]–[42]. Most of these works are based on context-based genetics and feedbacks between species' niche constructing traits and environments which often cause novel evolutionary trajectories [2], [43]. Our work here adds to the growing body of evidence criticizing species distribution models which assume that the environment alone can determine species distributions.

Not only should we consider the role of genetic diversity in species' adaptation to environmental heterogeneity [25] but also its role in affecting the ability and outcomes of niche construction on heterogeneous environments. Firstly, environmental gradients are necessary for the formation of stratified genotypic diversity (Fig.1). That is, environmental heterogeneity is a prerequisite for maintaining stable genetic variation. Secondly, band-like patterns of genotypic distribution only formed when the fitness is resource or context dependent. The stronger the resource-dependent component is relative to the fixed-fitness component in the fitness, the more bands will likely occur (Fig.4E & 5C). The strength of niche construction alone, through affecting the resource-dependent component in the fitness, can change the evolutional direction and genetic structures. Thirdly, the fitness landscape forms a valley at which the transition of genetic composition happens along the environmental gradient. This fitness valley will disappear when the selection is not acting on the gene loci affecting the niche-constructing ability (Fig.3B, 3D &3F).

Focusing on niche construction could provide new insights into biological conservation. Meffe and Carroll [44] emphasized the necessity for conservation biologists to take an evolutionary perspective, while niche construction is an underappreciated evolutionary process in shaping local environments and ecosystems [1], [3]. Traditional conservation planning works only on available genetic resources which are rapidly changing in this era of Anthropocene [45], often facing an increasingly bleak future [3]. When niche-constructing organisms cause physical changes in abiotic environments, these changes could become evolutionarily significant to other species due to modified selection pressure [1]. Jones et al. [46] envisaged defining the utility of ecosystem engineering in conservation, especially when those key engineering or niche-constructing species can be pre-identified (see also [1]). Here, our results emphasize the need to preserve environmental gradients for essential or limiting resources.

Our results raise questions on the effectiveness of using species distribution models (SDMs) for predicting species' potential range in novel environment, and forecasting range shift due to environmental changes. Four assumptions are typical of a SDM [47]–[48]: (i) species current distribution is at equilibrium, (ii) the fitted relationship between species known occurrence and habitat characteristics is an adequate representation of the realized niche, (iii) this relationship of the realized niche does not change across space and time (known as the niche conservatism; [49]), and (iv) species can access all niches via dispersal. Limited dispersal capacity and time window prohibit species to access remote and isolated niches, questioning the first and last assumptions [50]–[51], and the hybrid model which implements a dynamic process of spreading has been proposed as a remedy [28], [52]–[55]. Our results here further raise questions on assumptions (ii) and (iii), as the realized niche is often mediated by context-specific biotic interactions, with no sufficient evidence supporting a constant niche [50], [56]–[57]. In particular, the crux of niche construction is the coevolution of organisms and their environments, often causing a positive feedback known as the ecological inheritance. The observed distribution of a species is resultant from a long-term coupling of the species and its habitat, often extending beyond the bounds of its niche; that is, species can persist in areas where it cannot invade. As such, when projected in novel environments, the species potential distribution is likely inflated [1], [58]. Some introduced species can eventually establish and become invasive through the reinforcing feedback facilitated by humans, resulting in a regime shift in the recipient ecosystem [59]. Our results further showed that the positive feedback of niche construction can form sharp boundaries and a rapid transition of genetic composition along smooth environmental gradients, whereas slight changes in the slope of the environmental gradient can lead to drastic changes in species distribution. Therefore, not only can the environment affect species distribution, but also the environmental gradient. To this end, environmental homogenization and fragmentation will likely affect species distribution strongly; this has been ignored in current SDMs.

Individual-based models (IBMs) are a power tool to examine complex dynamic behavior and emerging patterns [60], and have increasingly been used in ecological studies [48]. Our model assumes that the individual of a focal cell selects the fittest neighbors to mate. As the fitness is determined by altered environment from past activities of niche constructing genotypes, we are essentially dealing with the process of ecological inheritance in a standard evolutionary model [1]. Our model has four limitations and can be expanded in future work. First, we simulated the spatial interaction using only the Von Neumann neighborhood, meaning that the gene flow only happens locally, therefore ignoring the potential effect of long-distance dispersal [24]. Along an environmental gradient, only the fittest has the opportunity for reproduction, therefore an individual that disperses away from the current optimal habitat will likely land itself in a suboptimal environment, and thus long-distance dispersal is selected against in our model. How long-distance dispersal affects the coupling of organisms and their environment is yet to be explored. Second, our model depicts the scenario of frequency-dependent selection in a zero-sum community [12]. Of course, as fitness can surely affect population demographics, considering a density-dependent selection could be more realistic and beget richer evolutionary dynamics [9]-[10], [61]. Third, our model only considers the feedback of a species' niche construction on its own fitness and distribution. A more realistic scenario could involve multiple species that affect each other through niche construction [10], [18]. Finally, the current model can be further expanded by allowing a time lag between the activity of niche construction and its impact on fitness. Such a time lag is often due to that sufficient resource change is needed for relevant genotypes to be selected, which can only be achieved through gradual and slow accumulation, forming evolutionary momentum and inertia [12], and complex spatial patterns (e.g. phase-lock oscillation; [62]).

Speciation most often happens via allopatric divergence where new species arise from reproduction isolation after separation by dispersal barriers. Doebeli & Dieckmann [63] offered a new theoretical perspective on the importance of environmental gradients to diversification through fostering frequency-dependent selection. The fitness valley identified here divides the distributions of high-fitness populations into two, with each consisting of unique genotypes. That is, intrinsically sympatric processes of organism-environment feedback can generate sharp geographical boundaries that separate genetically unique populations by the fitness valley. This fitness valley will further restrict potential gene flows between separated populations and thus promote allopatric speciation and diversification. The spatially local process of niche construction along a linear environmental gradient is capable of forming a reproduction barrier which facilitates speciation through genotype-environment feedback [15], [17]. Putting evolutionary processes into a spatially heterogeneous context with the organism and its environment co-affecting and co-adapting to each other could finally help to understand how future species will survive and adapt in the era of Anthropocene [64].
